# 4-[(*E*)-(5-Bromo-2-hydroxy­phen­yl)methyl­ideneamino]benzene­sulfonamide

**DOI:** 10.1107/S1600536809036010

**Published:** 2009-09-09

**Authors:** Zahid H. Chohan, Hazoor A. Shad, M. Nawaz Tahir

**Affiliations:** a Department of Chemistry, Bahauddin Zakariya University, Multan 60800, Pakistan; b Department of Physics, University of Sargodha, Sargodha, Pakistan

## Abstract

In the title compound, C_13_H_11_ClN_2_O_3_S, the dihedral angle between the benzene rings is 12.26 (33)° and an intra­molecular O—H⋯N hydrogen bond helps to establish the conformation. In the crystal, N—H⋯O and C—H⋯O inter­actions link the mol­ecules.

## Related literature

For a related structure and background discussion, see: Chohan *et al.* (2009 ▶). For graph-set theory, see: Bernstein *et al.* (1995 ▶).
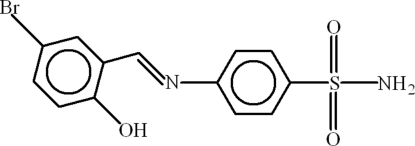


## Experimental

### 

#### Crystal data

C_13_H_11_BrN_2_O_3_S
*M* *_r_* = 355.21
Monoclinic, 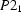

*a* = 6.1224 (15) Å
*b* = 4.5263 (13) Å
*c* = 23.445 (9) Å
β = 94.44 (2)°
*V* = 647.8 (3) Å^3^
*Z* = 2
Mo *K*α radiation
μ = 3.34 mm^−1^
*T* = 100 K
0.22 × 0.20 × 0.16 mm

#### Data collection

Bruker Kappa APEXII CCD diffractometer
Absorption correction: multi-scan (*SADABS*; Bruker, 2005 ▶) *T*_min_ = 0.482, *T*_max_ = 0.587
3181 measured reflections
1653 independent reflections
1458 reflections with *I* > 2σ(*I*)
*R*_int_ = 0.042

#### Refinement

*R*[*F* ^2^ > 2σ(*F* ^2^)] = 0.037
*wR*(*F* ^2^) = 0.087
*S* = 1.00
1653 reflections
188 parameters
1 restraint
H atoms treated by a mixture of independent and constrained refinement
Δρ_max_ = 0.71 e Å^−3^
Δρ_min_ = −1.13 e Å^−3^
Absolute structure: Flack (1983 ▶), 279 Friedal pairs
Flack parameter: 0.025 (16)

### 

Data collection: *APEX2* (Bruker, 2007 ▶); cell refinement: *SAINT* (Bruker, 2007 ▶); data reduction: *SAINT*; program(s) used to solve structure: *SHELXS97* (Sheldrick, 2008 ▶); program(s) used to refine structure: *SHELXL97* (Sheldrick, 2008 ▶); molecular graphics: *ORTEP-3 for Windows* (Farrugia, 1997 ▶) and *PLATON* (Spek, 2009 ▶); software used to prepare material for publication: *WinGX* (Farrugia, 1999 ▶) and *PLATON*.

## Supplementary Material

Crystal structure: contains datablocks global, I. DOI: 10.1107/S1600536809036010/hb5090sup1.cif

Structure factors: contains datablocks I. DOI: 10.1107/S1600536809036010/hb5090Isup2.hkl

Additional supplementary materials:  crystallographic information; 3D view; checkCIF report

## Figures and Tables

**Table 1:** Hydrogen-bond geometry (Å, °)

*D*—H⋯*A*	*D*—H	H⋯*A*	*D*⋯*A*	*D*—H⋯*A*
O1—H1⋯N1	0.82	1.86	2.583 (7)	146
N2—H21⋯O3^i^	0.73 (8)	2.26 (7)	2.950 (7)	160 (8)
N2—H22⋯O2^ii^	0.92 (7)	2.58 (8)	3.325 (7)	139 (6)
C9—H9⋯O2^iii^	0.93	2.57	3.386 (7)	146

Symmetry codes: (i) 
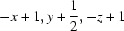
; (ii) 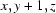; (iii) 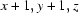.

**Fig. 1.**
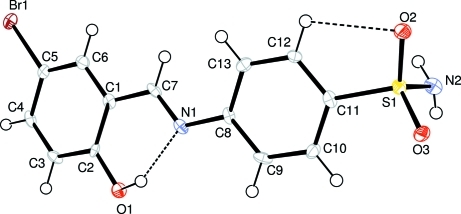
View of (I) with displacement ellipsoids drawn at the 50% probability level. H-atoms are shown by small circles of arbitrary radius. The dotted line indicate the intramolecular H-bond.

**Fig. 2.**
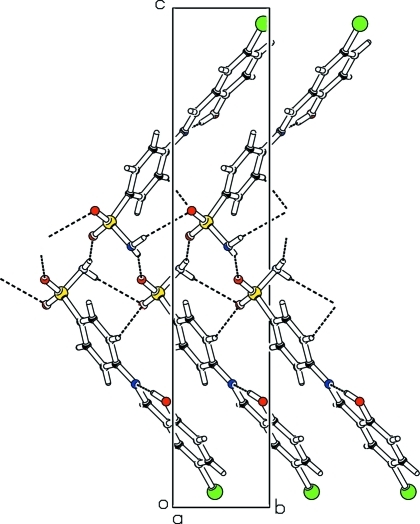
The partial packing of (I) showing how molecules form polymeric chains extending along the crystallographic b axis and ring motifs exist.

## supplementary crystallographic information

### Comment

As part of our ongoing studies of sulfonamides, we now report the structure of the title compound, (I). The crystal structure of (II) 4-{[(*E*)-(5-chloro-2-hydroxyphenyl) methylidene]amino}benzenesulfonamide (Chohan *et al.*, 2009) has been reported which differs from (I) due to chloro substitution instead of bromo.

In (I), the benzene rings A (C1—C6) of 5-bromosalicylaldehyde and B (C8—C13) of sulfanilamide are oriented at a dihedral angle of 12.03 (36)°. The Br1 and S1 atoms are at a distance of -0.016 (8) and -0.159 (9) A° from the mean square planes of rings A and B, respectively. There exist two intramolecular H-bonds (Table 1, Fig. 1) forming S(5) and S(6) ring motifs (Bernstein *et al.*, 1995). Three intermolecular H-bondings (Table 1) link the molecules in polymeric nature extending along the *b* axis (Fig. 2) and forming *R*_3_^3^(10) and *R*_2_^1^(8) ring motifs.

### Experimental

Sulfanilamide (0.344 g, 2 mmol) in ethanol (20 ml) was mixed with 5-bromosalicylaldehyde (0.402 g, 2 mmol) in ethanol (10 ml). The resultant mixture was refluxed for 4 h by monitoring through TLC. During refluxing the solution turned from colorless to orange yellow. After completion of reaction, it was cooled to room temperature, filtered and volume reduced to about one-third using rotary evaporator. It was then allowed to stand for 7 days at room temperature. After which a crystallized product was formed that was filtered, washed with ethanol (2 × 5 ml), dried and recrystallized in a mixture of methanol and ethanol (1:1) to afford shiny orange yellow prisms of (I).

### Refinement

The coordinates of H-atoms of the NH_2_ group were refined. The other H-atoms were positioned geometrically with O—H = 0.82, C—H = 0.93 Å for aromatic H atoms and constrained to ride on their parent atoms, with *U*_iso_(H) = xUeq(C, N, O), where *x* = 1.2 for all H atoms.

### Crystal data

**Table:**

C_13_H_11_BrN_2_O_3_S	*F*(000) = 356
*M**_r_* = 355.21	*D*_x_ = 1.821 Mg m^−^^3^
Monoclinic, *P*2_1_	Mo *K*α radiation, λ = 0.71073 Å
Hall symbol: P 2yb	Cell parameters from 1653 reflections
*a* = 6.1224 (15) Å	θ = 3.3–25.5°
*b* = 4.5263 (13) Å	µ = 3.34 mm^−^^1^
*c* = 23.445 (9) Å	*T* = 100 K
β = 94.44 (2)°	Prismatic, yellow
*V* = 647.8 (3) Å^3^	0.22 × 0.20 × 0.16 mm
*Z* = 2	

### Data collection

**Table:**

Bruker Kappa APEXII CCD diffractometer	1653 independent reflections
Radiation source: fine-focus sealed tube	1458 reflections with *I* > 2σ(*I*)
graphite	*R*_int_ = 0.042
Detector resolution: 7.80 pixels mm^-1^	θ_max_ = 25.5°, θ_min_ = 3.3°
ω scans	*h* = −6→7
Absorption correction: multi-scan (*SADABS*; Bruker, 2005)	*k* = −2→5
*T*_min_ = 0.482, *T*_max_ = 0.587	*l* = −28→28
3181 measured reflections	

### Refinement

**Table:**

Refinement on *F*^2^	Secondary atom site location: difference Fourier map
Least-squares matrix: full	Hydrogen site location: inferred from neighbouring sites
*R*[*F*^2^ > 2σ(*F*^2^)] = 0.037	H atoms treated by a mixture of independent and constrained refinement
*wR*(*F*^2^) = 0.087	*w* = 1/[σ^2^(*F*_o_^2^) + (0.0554*P*)^2^] where *P* = (*F*_o_^2^ + 2*F*_c_^2^)/3
*S* = 1.00	(Δ/σ)_max_ = 0.001
1653 reflections	Δρ_max_ = 0.71 e Å^−^^3^
188 parameters	Δρ_min_ = −1.13 e Å^−^^3^
1 restraint	Absolute structure: Flack (1983), 279 Friedal pairs
Primary atom site location: structure-invariant direct methods	Flack parameter: 0.025 (16)

### Special details

**Table:**

Geometry. Bond distances, angles *etc*. have been calculated using the rounded fractional coordinates. All su's are estimated from the variances of the (full) variance-covariance matrix. The cell e.s.d.'s are taken into account in the estimation of distances, angles and torsion angles
Refinement. Refinement of *F*^2^ against ALL reflections. The weighted *R*-factor *wR* and goodness of fit *S* are based on *F*^2^, conventional *R*-factors *R* are based on *F*, with *F* set to zero for negative *F*^2^. The threshold expression of *F*^2^ > σ(*F*^2^) is used only for calculating *R*-factors(gt) *etc*. and is not relevant to the choice of reflections for refinement. *R*-factors based on *F*^2^ are statistically about twice as large as those based on *F*, and *R*- factors based on ALL data will be even larger.

### Fractional atomic coordinates and isotropic or equivalent isotropic displacement parameters (Å^2^)

**Table:**

	*x*	*y*	*z*	*U*_iso_*/*U*_eq_	
Br1	0.22142 (8)	1.43584 (16)	0.03133 (2)	0.0184 (2)	
S1	0.2382 (2)	−0.1521 (3)	0.42877 (6)	0.0131 (4)	
O1	0.8682 (5)	0.9320 (15)	0.21201 (14)	0.0184 (11)	
O2	0.0460 (7)	−0.3040 (10)	0.40583 (17)	0.0175 (12)	
O3	0.4146 (6)	−0.3217 (10)	0.45633 (17)	0.0148 (12)	
N1	0.5538 (8)	0.6166 (11)	0.2472 (2)	0.0141 (16)	
N2	0.1639 (9)	0.0737 (13)	0.4770 (2)	0.0166 (17)	
C1	0.5019 (8)	0.947 (2)	0.16824 (19)	0.0124 (14)	
C2	0.7192 (9)	1.0429 (15)	0.1723 (2)	0.0134 (16)	
C3	0.7829 (9)	1.2590 (14)	0.1347 (2)	0.0148 (17)	
C4	0.6381 (9)	1.3763 (12)	0.0932 (2)	0.0143 (19)	
C5	0.4222 (9)	1.2702 (14)	0.0883 (2)	0.0120 (17)	
C6	0.3552 (10)	1.0646 (14)	0.1257 (2)	0.0149 (17)	
C7	0.4239 (9)	0.7346 (14)	0.2085 (2)	0.0134 (17)	
C8	0.4747 (8)	0.4182 (18)	0.2878 (2)	0.0114 (14)	
C9	0.6126 (9)	0.3535 (13)	0.3358 (2)	0.0147 (19)	
C10	0.5457 (10)	0.1737 (14)	0.3780 (3)	0.0155 (17)	
C11	0.3363 (9)	0.0532 (14)	0.3724 (2)	0.0135 (17)	
C12	0.1995 (9)	0.1113 (13)	0.3242 (2)	0.0134 (17)	
C13	0.2664 (9)	0.2936 (14)	0.2818 (2)	0.0145 (17)	
H1	0.81278	0.79686	0.22919	0.0218*	
H3	0.92711	1.32519	0.13772	0.0176*	
H4	0.68212	1.52308	0.06879	0.0172*	
H6	0.21007	1.00207	0.12277	0.0178*	
H7	0.27623	0.68391	0.20607	0.0155*	
H9	0.75293	0.43345	0.33940	0.0177*	
H10	0.63936	0.13250	0.41016	0.0182*	
H12	0.06076	0.02660	0.32024	0.0159*	
H13	0.17312	0.33314	0.24953	0.0172*	
H21	0.258 (12)	0.139 (18)	0.493 (3)	0.0197*	
H22	0.062 (11)	0.200 (17)	0.460 (3)	0.0197*	

### Atomic displacement parameters (Å^2^)

**Table:**

	*U*^11^	*U*^22^	*U*^33^	*U*^12^	*U*^13^	*U*^23^
Br1	0.0216 (3)	0.0200 (3)	0.0131 (3)	0.0009 (4)	−0.0012 (2)	0.0024 (3)
S1	0.0143 (7)	0.0109 (8)	0.0142 (7)	−0.0014 (6)	0.0010 (5)	0.0006 (5)
O1	0.0173 (18)	0.019 (2)	0.0186 (18)	−0.009 (3)	0.0003 (14)	0.010 (3)
O2	0.017 (2)	0.017 (2)	0.018 (2)	−0.006 (2)	−0.0015 (17)	0.0027 (19)
O3	0.014 (2)	0.013 (2)	0.017 (2)	0.0027 (18)	−0.0014 (17)	0.0053 (17)
N1	0.013 (2)	0.013 (3)	0.017 (3)	−0.002 (2)	0.005 (2)	−0.001 (2)
N2	0.018 (3)	0.010 (3)	0.022 (3)	−0.001 (3)	0.003 (2)	−0.001 (2)
C1	0.015 (2)	0.010 (3)	0.013 (2)	0.005 (4)	0.0056 (19)	−0.006 (4)
C2	0.017 (3)	0.018 (3)	0.005 (2)	−0.001 (3)	0.000 (2)	−0.003 (2)
C3	0.011 (3)	0.011 (3)	0.023 (3)	−0.002 (3)	0.006 (2)	0.004 (3)
C4	0.020 (3)	0.008 (4)	0.016 (3)	−0.005 (3)	0.009 (2)	0.001 (2)
C5	0.016 (3)	0.010 (3)	0.010 (3)	0.005 (3)	0.002 (2)	−0.006 (2)
C6	0.015 (3)	0.015 (3)	0.015 (3)	−0.001 (3)	0.003 (2)	−0.004 (3)
C7	0.013 (3)	0.008 (3)	0.020 (3)	−0.004 (3)	0.007 (2)	−0.002 (3)
C8	0.017 (2)	0.004 (3)	0.014 (2)	0.002 (3)	0.006 (2)	0.001 (3)
C9	0.009 (3)	0.010 (4)	0.025 (3)	−0.002 (2)	0.001 (2)	−0.002 (2)
C10	0.017 (3)	0.016 (3)	0.013 (3)	0.001 (3)	−0.002 (2)	0.003 (3)
C11	0.017 (3)	0.008 (3)	0.016 (3)	0.001 (3)	0.005 (2)	0.001 (2)
C12	0.012 (3)	0.014 (3)	0.014 (3)	−0.004 (3)	0.000 (2)	−0.001 (3)
C13	0.016 (3)	0.013 (3)	0.014 (3)	0.002 (3)	−0.002 (2)	−0.002 (3)

### Geometric parameters (Å, °)

**Table:**

Br1—C5	1.898 (5)	C4—C5	1.403 (8)
S1—O2	1.431 (5)	C5—C6	1.363 (8)
S1—O3	1.437 (4)	C8—C13	1.392 (8)
S1—N2	1.616 (5)	C8—C9	1.385 (7)
S1—C11	1.759 (6)	C9—C10	1.368 (9)
O1—C2	1.349 (7)	C10—C11	1.390 (8)
O1—H1	0.8200	C11—C12	1.379 (7)
N1—C8	1.421 (8)	C12—C13	1.378 (8)
N1—C7	1.276 (7)	C3—H3	0.9300
N2—H21	0.73 (8)	C4—H4	0.9300
N2—H22	0.92 (7)	C6—H6	0.9300
C1—C7	1.454 (9)	C7—H7	0.9300
C1—C6	1.395 (8)	C9—H9	0.9300
C1—C2	1.396 (8)	C10—H10	0.9300
C2—C3	1.393 (8)	C12—H12	0.9300
C3—C4	1.372 (7)	C13—H13	0.9300
			
O2—S1—O3	118.7 (3)	C9—C8—C13	119.5 (5)
O2—S1—N2	107.4 (3)	N1—C8—C9	117.4 (5)
O2—S1—C11	106.9 (3)	C8—C9—C10	121.0 (5)
O3—S1—N2	105.4 (3)	C9—C10—C11	119.4 (6)
O3—S1—C11	109.4 (3)	S1—C11—C10	120.2 (4)
N2—S1—C11	108.8 (3)	C10—C11—C12	120.0 (5)
C2—O1—H1	109.00	S1—C11—C12	119.7 (4)
C7—N1—C8	121.0 (5)	C11—C12—C13	120.6 (5)
S1—N2—H22	108 (5)	C8—C13—C12	119.4 (5)
S1—N2—H21	111 (6)	C2—C3—H3	119.00
H21—N2—H22	117 (8)	C4—C3—H3	119.00
C2—C1—C6	119.2 (6)	C3—C4—H4	121.00
C2—C1—C7	121.4 (5)	C5—C4—H4	121.00
C6—C1—C7	119.4 (5)	C1—C6—H6	120.00
O1—C2—C1	121.4 (5)	C5—C6—H6	120.00
C1—C2—C3	119.2 (5)	N1—C7—H7	119.00
O1—C2—C3	119.4 (5)	C1—C7—H7	119.00
C2—C3—C4	121.6 (5)	C8—C9—H9	119.00
C3—C4—C5	118.6 (5)	C10—C9—H9	119.00
C4—C5—C6	120.6 (5)	C9—C10—H10	120.00
Br1—C5—C4	118.5 (4)	C11—C10—H10	120.00
Br1—C5—C6	120.8 (4)	C11—C12—H12	120.00
C1—C6—C5	120.7 (6)	C13—C12—H12	120.00
N1—C7—C1	121.4 (5)	C8—C13—H13	120.00
N1—C8—C13	123.1 (5)	C12—C13—H13	120.00
			
O2—S1—C11—C10	−166.2 (5)	O1—C2—C3—C4	179.3 (5)
O2—S1—C11—C12	18.2 (6)	C1—C2—C3—C4	−1.1 (9)
O3—S1—C11—C10	−36.6 (6)	C2—C3—C4—C5	−1.2 (8)
O3—S1—C11—C12	147.9 (5)	C3—C4—C5—Br1	179.4 (4)
N2—S1—C11—C10	78.1 (6)	C3—C4—C5—C6	3.0 (8)
N2—S1—C11—C12	−97.5 (5)	Br1—C5—C6—C1	−178.8 (5)
C8—N1—C7—C1	177.5 (6)	C4—C5—C6—C1	−2.5 (9)
C7—N1—C8—C9	−165.3 (6)	N1—C8—C9—C10	177.4 (6)
C7—N1—C8—C13	13.4 (10)	C13—C8—C9—C10	−1.3 (10)
C6—C1—C2—O1	−178.8 (6)	N1—C8—C13—C12	−177.7 (6)
C6—C1—C2—C3	1.6 (10)	C9—C8—C13—C12	1.0 (9)
C7—C1—C2—O1	3.2 (10)	C8—C9—C10—C11	0.3 (9)
C7—C1—C2—C3	−176.4 (6)	C9—C10—C11—S1	−174.5 (5)
C2—C1—C6—C5	0.2 (10)	C9—C10—C11—C12	1.1 (9)
C7—C1—C6—C5	178.2 (6)	S1—C11—C12—C13	174.2 (5)
C2—C1—C7—N1	−3.7 (10)	C10—C11—C12—C13	−1.4 (9)
C6—C1—C7—N1	178.3 (6)	C11—C12—C13—C8	0.4 (9)

### Hydrogen-bond geometry (Å, °)

**Table:**

*D*—H···*A*	*D*—H	H···*A*	*D*···*A*	*D*—H···*A*
O1—H1···N1	0.82	1.86	2.583 (7)	146
N2—H21···O3^i^	0.73 (8)	2.26 (7)	2.950 (7)	160 (8)
N2—H22···O2^ii^	0.92 (7)	2.58 (8)	3.325 (7)	139 (6)
C9—H9···O2^iii^	0.93	2.57	3.386 (7)	146

Symmetry codes: (i) −*x*+1, *y*+1/2, −*z*+1; (ii) *x*, *y*+1, *z*; (iii) *x*+1, *y*+1, *z*.
